# Myxoid Chondrosarcoma of Maxilla in a Pediatric Patient: A Rare Case Report

**DOI:** 10.1155/2016/5419737

**Published:** 2016-01-24

**Authors:** Pranali Nimonkar, Nitin Bhola, Anendd Jadhav, Anuj Jain, Rajiv Borle, Rajul Ranka, Minal Chaudhary

**Affiliations:** ^1^Department of Oral and Maxillofacial Surgery, Sharad Pawar Dental College and Hospital, Datta Meghe Institute of Medical Sciences, Sawangi, Wardha, Maharashtra 442004, India; ^2^Department of Oral Pathology and Microbiology, Sharad Pawar Dental College and Hospital, Datta Meghe Institute of Medical Sciences, Sawangi, Wardha, Maharashtra 442004, India

## Abstract

Myxoid variant of chondrosarcoma is an uncommon potentially lethal malignant tumor which is even rare in pediatric age group. In the present paper, we report one such case of intermediate grade myxoid chondrosarcoma of left side of maxilla in a 12-year-old girl. The present case had a firm, painless, and lobulated growth in premolar-molar region which was associated with bicortical expansion. Maxillofacial imaging showed ill-defined radiolucency with displaced maxillary molars. Osteolytic changes were evident with the alveolus and walls of maxillary sinus. Owing to the age of the patient, surgical excision was selected as the modality of management followed by postoperative radiotherapy. This report encompasses the entire gamut of clinicopathological, radiological, and treatment modalities employed for chondrosarcoma.

## 1. Introduction

Chondrosarcoma is an uncommon, slowly enlarging, malignant tumor which has its origin from cartilaginous tissue or bone derived from chondroid precursors, resulting in abnormal bone and/or cartilage growth [1]. It represents the second largest group of bone tumors after osteosarcoma [2], with less than 10% of cases in head and neck region [3] and accounting for 0.1% of all malignant tumors in this region. This tumor usually grows within a bone or on its surface [4] affecting any bone but shows prevalence for pelvic girdle, chest wall, and scapula [5]. In head and neck region, the most common sites of occurrence are larynx, thyroid cartilage, and arytenoids [6]. However, chondrosarcomas can occur in all other sites of craniofacial compartment in which cartilage is found such as maxilla, mandible, paranasal sinuses, nasopharynx, and base of skull [7]. In the head and neck, chondrosarcomas are slightly more common in men than in women and primarily occur in the third to sixth decade of life [4]. It is found that chondrosarcoma is usually more aggressive in younger individuals than in adults [8]. Since this tumor is rare and aggressive in younger individuals, development of therapeutic standards in this complex anatomy of head and neck region depends on individual case.

In this report, we present a case of a 12-year-old girl with myxoid chondrosarcoma involving maxilla and review the clinical presentation, histopathology, and treatment of the same.

## 2. Case Report

A 12-year-old girl was referred to the Department of Oral and Maxillofacial Surgery, Sharad Pawar Dental College, Datta Meghe Institute of Medical Sciences, in January 2015 for evaluation of painless growth on the left side of her maxilla. The patient and her mother had noticed the mass approximately 4 months earlier. The medical and family histories were unremarkable.

**Figure 1:**
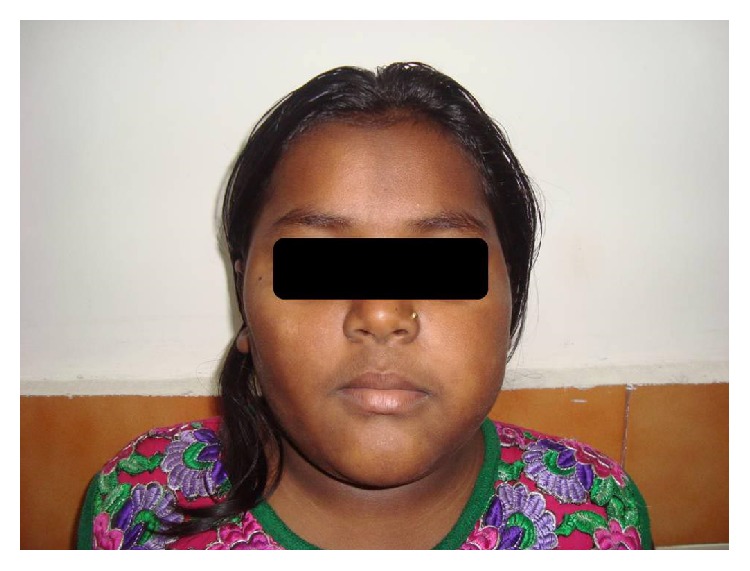
Extraoral photograph showing asymmetry.

There was facial asymmetry caused by a mass within the left buccal area (Figure 1).

**Figure 2:**
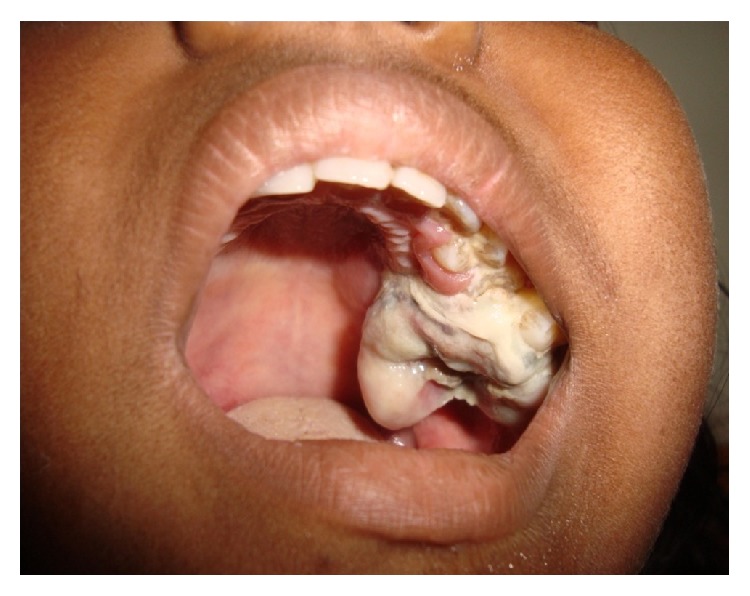
Intraoral growth on left side of maxilla.

There was no cervical lymphadenopathy. Intraoral examination revealed the presence of a lobular maxillary growth measuring approximately 4.0 × 3.0 cm in size extending from the second premolar to the maxillary tuberosity in buccopalatal aspect of left side. The regional teeth were displaced and mobile (Figure 2).

On palpation, it was firm and slightly tender and was associated with expansion of both cortical plates. Egg shell crackling was evident over some areas of buccal cortical plate.

**Figure 3:**
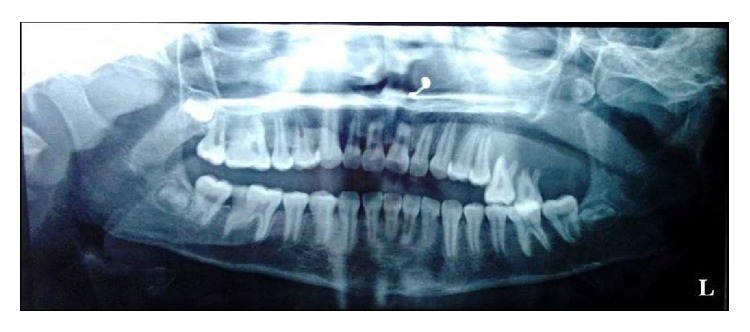
Orthopantomogram showing displaced molars with resorption of roots.

Radiological examination showed radiolucency displacing the molars along with resorption of roots (Figure 3).

**Figure 4:**
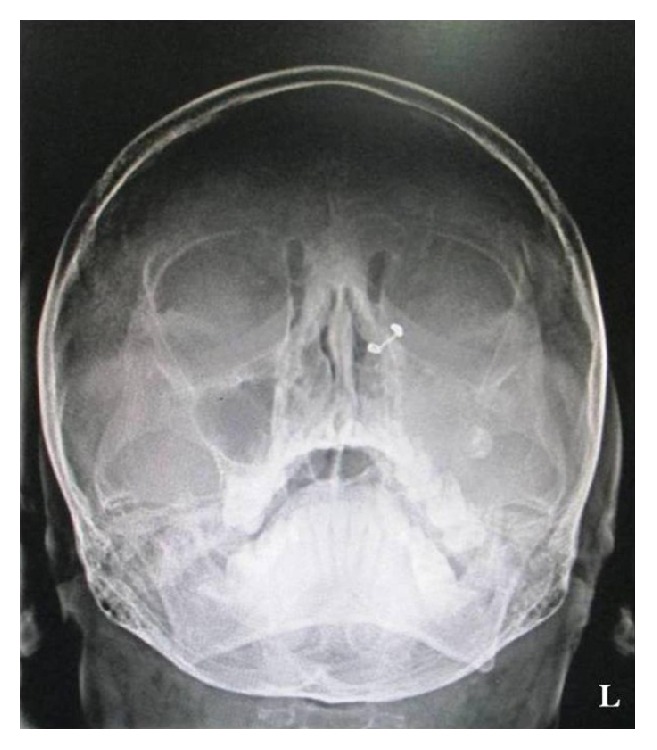
PNS view showing destruction of floor of maxillary antrum on left side.

Water's view showed destruction of floor of maxillary sinus with complete haziness of the sinus and ill-defined borders. Superior displacement of impacted third molar was also seen (Figure 4).

**Figure 5:**
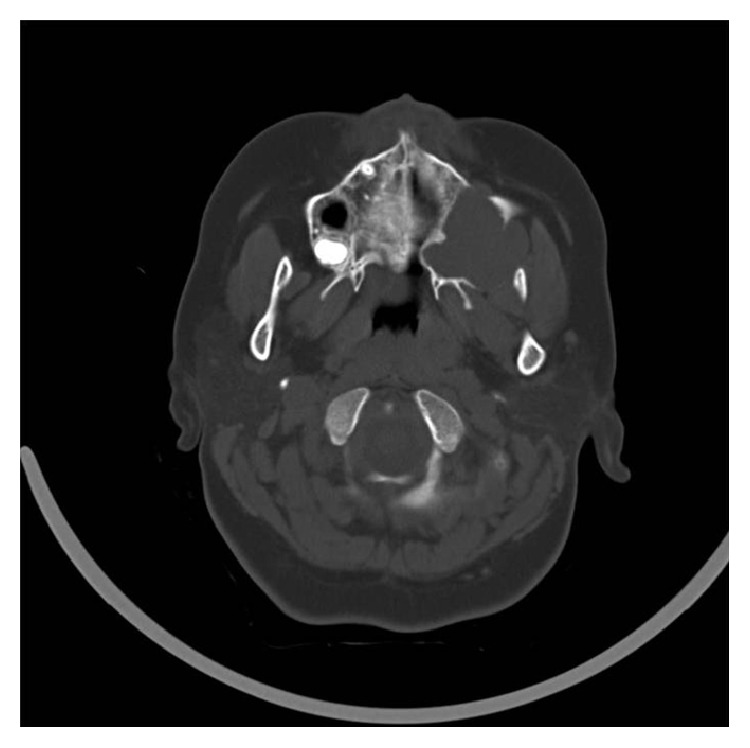
Axial section of CT scan showing osteolytic changes.

**Figure 6:**
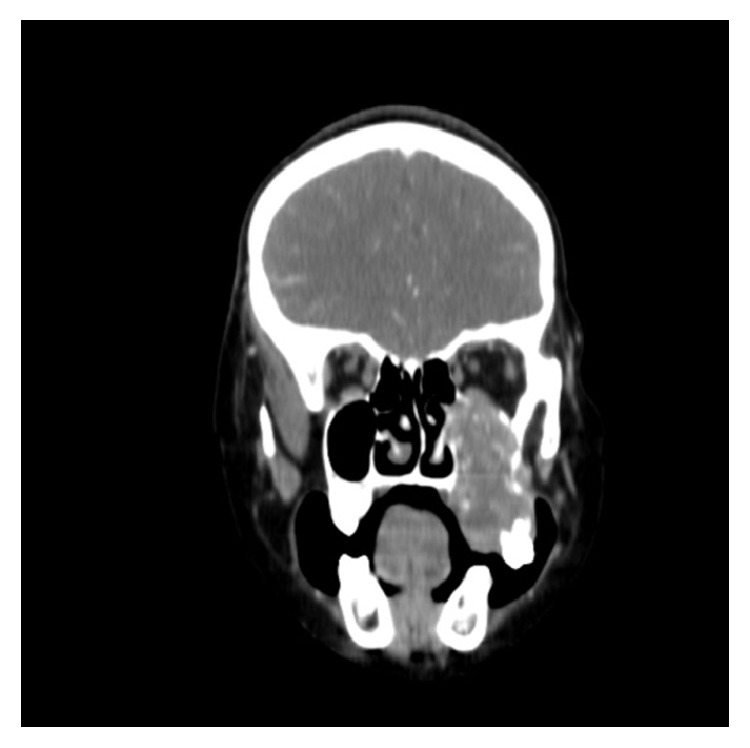
Coronal section of CT scan showing extent of tumor.

Computed Tomography (CT) imaging showed an irregular soft tissue mass causing osteolytic destruction of upper left maxillary alveolus, floor, medial wall, and lateral wall of left maxillary sinus (Figures 5 and 6).

Since the lesion was aggressive, malignant neoplasms were considered in differential diagnosis. Salivary gland malignancies like mucoepidermoid carcinoma and adenoid cystic carcinoma were primarily considered as they occur commonly on palate. Both of these lesions do not cause bicortical expansion which was present in our case. Carcinoma of maxillary sinus was also included in differential diagnosis although it is typically a disease of adults and is associated with habit history. Malignant mesenchymal tumors like osteosarcoma and chondrosarcoma, though rare in craniofacial region, were considered in differential diagnosis. Another group of lesions like Hodgkin's and non-Hodgkin's lymphoma can present as mass in the palate with ulceration but are seldom reported. Moreover, lymphomas most frequently present as cervical lymphadenopathy which was absent in the present case.

Incisional biopsy was performed under local anesthesia, and the specimen was subjected to histopathologic evaluation. Microscopic examination revealed a hypercellular connective tissue stroma comprising abundant cartilage, a lobulated growth pattern with round and oval cells in lacunae showing nuclear pleomorphism, nuclear atypia, and hyperchromasia. Mitotic activity was mild and at places large plump chondroblasts and binucleated chondrocytes were seen. Large loose basophilic areas were seen in connective tissue suggestive of myxoid stroma. The final diagnosis was made as intermediate grade myxoid chondrosarcoma of left maxilla.

**Figure 7:**
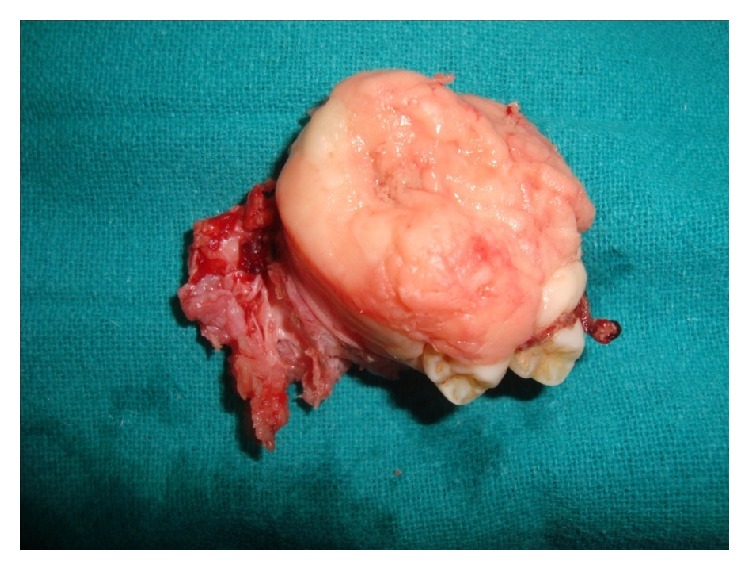
Resected tumor mass.

Under general anesthesia, degloving incision was given in left maxillary buccal vestibule and surgical excision of tumor was done (Figure 7).

**Figure 8:**
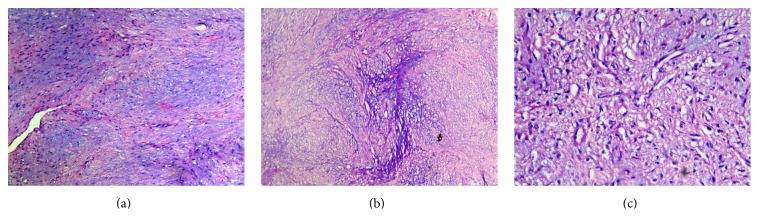
(a) Fibrous stroma with myxoid component (10x). (b) Chondroid tissue (10x). (c) Chondrocytes showing nuclear atypia, hyperchromasia, and pleomorphism (40x).

The surgical defect was closed with medicated gauze pack placed under palatal surgical splint. Posthealing palatal obturator was fabricated to prevent contamination. The removed mass was firm and rubbery in consistency and was sent for histopathologic evaluation which confirmed the preoperative diagnosis (Figure 8).

The patient was then subjected to postoperative radiotherapy because of the inadequate removal of tumor as maxillectomy was not performed considering the age of the patient. The patient is under followup and after 10 months she is disease-free with no signs of recurrence.

## 3. Discussion

Chondrosarcoma was considered to be a variant of osteosarcoma before 1930 when Pheimeister first described it as a separate entity [9]. The prevalence of this entity in jaws is controversial. In head and neck region, the most common site of occurrence differs according to various studies published. To be particular, the anterior portion of maxilla and the posterior region of the mandible are more prevalent locations of occurrence [10]. It can occur in any age but is usually found in adults between 3rd and 6th decades of life, although the youngest patient reported is 16 months old and the oldest one is a 74-year-old man [11]. Pediatric patients develop head and neck chondrosarcomas with even less frequency, although a few authors believe that the head and neck region accounts for a higher percentage of chondrosarcoma in children than in adults [11]. When all head and neck sites are considered, this entity has a male predilection with a ratio of 1.2 : 1 [3]. Myxoid chondrosarcoma is a rare histologic variant of chondrosarcoma. This variant rarely originates in head and neck region and is extremely rare in patients younger than 20 years of age. It also shows a high rate of local recurrence [12]. This case is of specific interest since a female pediatric patient was affected by the myxoid variant of chondrosarcoma in posterior maxillary region making it an exceptional case.

Chondrosarcoma has been considered to be a malignant tumor histogenetically derived from mature cartilaginous tissue. Since maxilla is a bone of exclusive membranous ossification, the possibility of chondrosarcoma is difficult to explain. However, it is thought to arise from vestigial cartilage remnants in periodontal ligament, cartilage found in incisive papilla, foci of cartilage from cartilaginous nasal capsule, and paraseptal cartilage. Thus, it explains the notion that chondrosarcoma in maxilla is derived from cartilaginous differentiation of primitive mesenchymal cells rather than from embryonic cartilaginous nests [13].

Unlike the expanding high grade chondrosarcoma of the long bones presenting with excruciating pain, the chondrosarcoma of head and neck tends to be painless on presentation. The common reported symptoms are swelling/mass (68%), nasal obstruction (32%), epistaxis (32%), and tooth mobility (24%) [14]. Progression of the mass may lead to other symptoms such as headache, blurred vision, proptosis, diplopia, and facial swelling. Rarely cervical lymphadenopathy may be evident [15]. Duration of signs and symptoms before diagnosis ranged from 2 weeks to 4 years [14]. Our case was presented and diagnosed after a period of 4 months with a painless swelling and tooth mobility.

A thorough radiological examination including periapical radiograph and panoramic radiograph as well as CT scan may provide clues to diagnosis. The conventional radiographic findings usually include irregular intramedullary radiolucencies interspersed with punctuate radiopacities, expansion and destruction of the cortical plates, PDL spaces widening, or even sunburst appearance at the periphery [2, 3, 16]. CT scan is superior in defining the peripheral extent of the neoplasm compared to panoramic radiographs [17]. Moreover, CT scan is more sensitive than MRI for detection of calcifications. CT scan may demonstrate an ill-defined cloud-like matrix with calcified whorls and arcs [6].

Diagnosis can only be established by histopathologic examination. Lichtenstein and Jaffe established a histopathological diagnostic criterion for chondrosarcoma. Histologically, chondrosarcoma can be classified according to the microscopic appearance into conventional, clear cell, myxoid, mesenchymal, and dedifferentiated [18]. Chondrosarcoma presents as a malignant tumor composed of fully developed cartilage without tumor osteoid, being directly formed from a sarcomatous stroma. Myxoid changes, calcifications, and ossifications may be seen [19]. Evans et al. in 1977 had histologically graded chondrosarcomas according to their degree of cellularity, atypia, mitotic activity, nuclear size, and surrounding matrix composition from grade I to grade III. This grading system is important because it reflects prognosis based on tumor biology distinct from its location or stage of presentation [16]. In our case, the histological appearance was conclusive of intermediate grade myxoid chondrosarcoma.

Because of the rarity of chondrosarcoma of the jaw, there are no established evidence based treatment protocols [6]. Chondrosarcoma is generally treated with multimodal approach, wide en-bloc resection [20], local curettage [21], cryotherapy [21], chemotherapy [22], radiotherapy [23], and immunotherapy [24]. Depending on age, sex, size, and extent of tumor, the treatment modality should be decided. Adequate surgical resection remains the gold standard for the treatment of chondrosarcoma of jaw [19]. Ideal initial resection includes bone margin of 2-3 cm surrounding the lesion [25]. Although the use of cryosurgery can be associated with complications such as infection, embolism, and neuropathy, it is suggested for the treatment of grade I chondrosarcoma [26]. Chondrosarcoma is believed to be radioresistant because of prolonged response time to irradiation [21]. However, radiotherapy could have a role in cases of incompletely resected or inoperable tumors [24]. The use of chemotherapy before or after surgery is controversial [6]. Chemotherapy as a neoadjuvant treatment inhibits the tumor growth and progression [6]. However, it is not beneficial in improving the long term survival or distant metastasis control. Some authors suggest that the combination of both radiotherapy and chemotherapy is synergistic in reducing the viability of tumor cells that may disperse during surgical procedures [27]. The surgical approach often requires extensive ablative procedures that can compromise major functional and esthetic elements and necessitates the performance of complex bone reconstructive techniques [20]. The intralesional excision-curettage of large lesions, combined with a powerful local adjuvant radiation therapy, can also be advocated in special situation [6]. In our case, we have opted for the intralesional excision-curettage of the lesion followed by radiation therapy owing to the pediatric age group of the patient.

Followup at regular intervals with repeated investigations may be necessary due to high recurrence rate and distant metastasis [28]. Local recurrences are quite frequent accounting for 20–60% of the cases. Chondrosarcomas can recur at any time ranging from a few months to several years after the initial diagnosis and treatment [2]. Approximately 20% of tumors metastasize, predominantly to the lungs. The prognosis is reported to be good for low and intermediate grade chondrosarcoma [29]. The five-year survival rate is 90% for grade I chondrosarcoma, 81% for grade II chondrosarcoma, and 43% for grade III chondrosarcoma [28]. So early detection may be beneficial to enhance the treatment and thus to improve the quality of life of the patient.

## 4. Conclusion

Myxoid chondrosarcoma is a rare entity in head and neck region of patients in pediatric age group. Early and accurate diagnosis of this tumor helps in formulating a proper treatment plan. Considering patient characteristics, the nature of tumor, and high recurrence rate, a proper treatment modality must be chosen. Long term regular followup is mandatory for such tumors.

## References

[1] Koch B. B., Karnell L. H., Huffman H. T. (2000). National cancer database report on chondrosarcoma of the head and neck. *Head & Neck*.

[2] Randall R., Hunt K. J. (2006). Chondrosarcoma of the bone. *ESUN—Liddy Shriver Sarcoma Initiative*.

[3] Chowdhury A., Kalsotra P., Bhagat D. R., Sharma P., Katoch P. (2008). Chondrosarcoma of the Maxilla-Recurrent. *JK Science*.

[4] Kundu S., Pal M., Paul R. (2011). Clinicopathologic correlation of chondrosarcoma of mandible with a case report. *Contemporary Clinical Dentistry*.

[5] Hackney F. L., Aragon S. B., Aufdemorte T. B., Holt G. R., Van Sickels J. E. (1991). Chondrosarcoma of the jaws: clinical findings, histopathology and treatment. *Oral Surgery, Oral Medicine, Oral Pathology*.

[6] Sammartino G., Marenzi G., Howard C. M. (2008). Chondrosarcoma of the jaw: a closer look at its management. *Journal of Oral and Maxillofacial Surgery*.

[7] Finn D. G., Goepfert H., Batsakis J. G. (1984). Chondrosarcomas of the head and neck. *Laryngoscope*.

[8] Huvos A. G., Marcove R. C. (1987). Chondrosarcoma in the young. A clinicopathologic analysis of 79 patients younger than 21 years of age. *The American Journal of Surgical Pathology*.

[9] Pheimeister D. B. (1930). Chondrosarcoma of bone. *Surgery Gynecology and Obstretics*.

[10] Huang C. C., Huang J. S., Wong T. Y., Chen K. C., Huang T. T. (2014). Chondrosarcoma of the Maxilla—a case report. *Taiwan Journal of Oral and Maxillofacial Surgery*.

[11] Lone S. A., Sajad M., Lateef M. (2003). Chondrosarcoma of the paranasal sinuses. *JK Science*.

[12] Antonescu C. R., Argani P., Erlandson R. A., Healey J. H., Ladanyi M., Huvos A. G. (1998). Skeletal and extraskeletal myxoid chondrosarcoma: a comparative clinicopathologic, ultrastructural, and molecular study. *Cancer*.

[13] Massarelli G., Gandolfo L., Tanda F., Ghiselli F., Manunta V. (1988). Maxillary chondrosarcoma. (Report of two cases). *The Journal of Laryngology & Otology*.

[14] Tien N., Chaisuparat R., Fernandes R. (2007). Mesenchymal Chondrosarcoma of the maxilla: case report and literature review. *Journal of Oral and Maxillofacial Surgery*.

[15] Ruark D. S., Schlehaider U. K., Shah J. P. (1992). Chondrosarcomas of the head and neck. *World Journal of Surgery*.

[16] Evans H. L., Ayala A. G., Romsdahl M. M. (1977). Prognostic factors in chondrosarcoma of bone: a clinicopathologic analysis with emphasis on histologic grading. *Cancer*.

[17] de Oliveira R. C., Marques K. D. S., de Mendonca A. R., da Silva M. R. B., Batista A. C., Ribeiro-Rotta R. F. (2009). Chondrosarcoma of the temporomandibular joint: a case report in a child. *Journal of Orofacial Pain*.

[18] Takahama A., Alves F. D. A., Prado F. O., Lopes M. A., Kowalski L. P. (2012). Chondrosarcoma of the maxilla: report of two cases with different behaviours. *Journal of Cranio-Maxillo-Facial Surgery*.

[19] Chauhan N. P., Pai K. M., Mutalik S., Balakrishnan R., Valiathan M., Sujir N. (2014). A progressively enlarging swelling of the palate. *Oral Surgery, Oral Medicine, Oral Pathology and Oral Radiology*.

[20] Oujilal A., el Alami M. N., Lazrak A., Jazouli N., Kzadri M. (2001). Chondrosarcoma of the jaw. A case localized to the mandible. *Revue de Stomatologie et de Chirurgie Maxillo-Faciale*.

[21] Aziz S. R., Miremadi A. R., McCabe J. C. (2002). Mesenchymal chondrosarcoma of the maxilla with diffuse metastasis: case report and literature review. *Journal of Oral and Maxillofacial Surgery*.

[22] Crawford J. G., Oda D., Egbert M., Myall R. (1995). Mesenchymal chondrosarcoma of the maxilla in a child. *Journal of Oral and Maxillofacial Surgery*.

[23] Burkey B. B., Hoffman H. T., Baker S. R., Thornton A. F., McClatchey K. D. (1990). Chondrosarcoma of the head and neck. *Laryngoscope*.

[24] Garrington G. E., Collett W. K. (1988). Chondrosarcoma. I. A selected literature review. *Journal of Oral Pathology*.

[25] Carlson E. R., Panella T., Holmes J. D. (2004). Sarcoma of mandible. *Journal of Oral and Maxillofacial Surgery*.

[26] Veth R., Schreuder B., van Beem H., Pruszczynski M., de Rooy J. (2005). Cryosurgery in aggressive, benign, and low-grade malignant bone tumours. *The Lancet Oncology*.

[27] Molla M. R., Ijuhin N., Sugata T., Sakamoto T. (1987). Chondrosarcoma of the jaw: report of two cases. *Journal of Oral and Maxillofacial Surgery*.

[28] Divyalakshmi M. R., Iyengar A. R., Nagesh K. S., Chhabra S., Ramneek (2012). Chondrosarcoma of the Maxilla: report of a rare case. *Annals of Dental Research*.

[29] Gallego L., Junquera L., Fresno M. F., de Vicente J. C. (2009). Chondrosarcoma of the temporomandibular joint. A case report and review of the literature. *Medicina Oral, Patologia Oral y Cirugia Bucal*.

